# The International Editorship of Freshwater Systems

**DOI:** 10.1100/tsw.2001.78

**Published:** 2001-09-13

**Authors:** Karl E. Havens

## Abstract

It is my pleasure to announce that two distinguished internationalscientists have joined the editorship of the FreshwaterSystems domain of TheScientificWorldJOURNAL — Professor BrijGopal of Jawaharlal Nehru University (India) and Dr. Manual Graça of the Universityof Coimbra (Portugal). Professor Gopal is the Secretary General of the NationalInstitute of Ecology, Editor of the InternationalJournal of Ecology & Environmental Science,and Chairman of the SIL (International Association of Theoretical and AppliedLimnology) Committee on Limnology in Developing Countries. His research interestsinclude the ecology, biogeochemistry and biodiversity of wetland ecosystems,the management of wetlands as an integral part of the watershed, and wetlandwater policy–related issues. Dr. Graça is a stream ecologist whose researchinterests include the two general areas of organic matter decomposition andbiological monitoring. His specific areas of research focus include quantificationof organic matter and other chemical changes in decomposing leaves, the ecologyof aquatic hyphomycetes, and the ecology of animals feeding on detritus. Hisresearch dealing with biological monitoring is carried out in close cooperationwith the paper and mining industries, facilitating the practical applicationof his work.

With the addition of Professor Gopal and Dr. Graça as Associate Editors, joining Professor Ramesh Reddy (University of Florida, U.S.), a broad range of expertise is represented in the scientific leadership of the *Freshwater Systems* domain. *Freshwater Systems* also has an Editorial Board comprised of 23 internationally known scientists who are leaders in their respective fields. A complete listing of the editors and board members may be found at the end of this editorial.

In its first year of production, several important review articles have been published under the *Freshwater Systems* domain, including a seminal review on cyanobacteria blooms by Dr. Hans Paerl (U.S.), a review of the concept of alternative stable states in shallow lakes by Dr. Martin Scheffer (the Netherlands), and a thought-provoking synthesis regarding biomanipulation of lakes with filter feeding fish by Professor Ping Xie (P.R. China). During the next year *Freshwater Systems* will continue this trend in publishing review papers on major issues in wetland, lake, and flowing water science, as well as original research articles. In the near future, the domain also will have a unique home page on the web site of TheScientificWorld, where freshwater scientists and resource managers can directly access information on the latest publications, biographical sketches of all board members and editors, instructions to authors, and the specific aims and scope of the journal. In the interim, information for prospective authors can be readily obtained from the home page of TheScientificWorldJOURNAL.

## FRESHWATER SYSTEMS – EDITORIAL BOARD

### PRINCIPAL EDITOR

**Dr. Karl E. Havens**
South Florida Water Management District, Florida, U.S.

### ASSOCIATE EDITORS

**Dr. Manuel A.S. Graça,** Departamento de Zoologia, Universidade de Coimbra, Portugal
**Professor Brij Gopal,** School of Environmental Sciences, Jawaharal Nehru University, India
**Professor K. Ramesh Reddy,** Soil and Water Science Department, University of Florida, U.S.

### EDITORIAL BOARD MEMBERS

#### *Lakes*

**Dr. Michael S. Allen,** Department of Fisheries and Aquatic Sciences, The University of Florida, U.S.
**Dr. Paul C. M. Boers,** Institute for Inland Water, The Netherlands
**Professor Richard W. Douglas,** School of Environmental Sciences, University of Ulster at Coleraine, Northern Ireland, U.K.
**Professor Takehiko Fukushima,** Department of Civil and Environmental Engineering, Hiroshima University, Japan
**Professor Ursula Gaedke,** Institut für Biochemie und Biologie, Universität Potsdam, Germany
**Professor Hans-Peter Grossart,** University of Oldenburg, Germany
**Professor Asit Mazumder,** Department of Biology, University of Victoria, Canada
**Dr. Marianne Moore,** Department of Biological Sciences, Wellesley College, U.S.
**Professor Bernadette Pinel-Alloul,** Departement de Sciences Biologiques, Universite de Montreal, Canada
**Professor Val H. Smith,** Department of Systematics and Ecology, University of Kansas, U.S.
**Dr. Ellen van Donk,** Netherlands Institute of Ecology, Center for Limnology, The Netherlands
**Professor Dr. Ping Xie,** Donghu Experimental Station of Lake Ecosystems, The Chinese Academy of Sciences, China

#### *Wetlands*

**Dr. Daniel L. Childers,** Florida International University, U.S.
**Professor Ron DeLaune,** Wetland Biogeochemistry Institute, Louisiana State University, U.S.
**Dr. Dale E. Gawlik,** South Florida Water Management District, U.S.
**Professor L. Gordon Goldsborough,** Department of Botany, University of Manitoba, Canada
**Dr. Michael R. Smart,** U.S. Army Corps of Engineers, Lewisville, Texas, U.S.

#### *Rivers & Streams*

**Dr. Joseph Culp**, National Water Research Institute, Environment Canada, Canada
**Dr. Laura Davalos-Lind,** Chapala Ecology Station, Baylor University, U.S.
**Professor David Dudgeon,** University of Hong Kong, China
**Professor Anne E. Hershey,** Biology Department, University of North Carolina, U.S.
**Dr. Gea-Jae Joo,** Department of Biology, Pusan National University, South Korea
**Professor Rex L. Lowe,** Department of Biological Sciences, Bowling Green State University, U.S.
